# Evidence and evidence gaps in therapies of nasal obstruction and rhinosinusitis

**DOI:** 10.3205/cto000133

**Published:** 2016-12-15

**Authors:** Nicole Rotter

**Affiliations:** 1 Department of Otolaryngology, Head and Neck Surgery, Ulm University Medical Centre, Ulm, Germany

**Keywords:** evidence-based therapies, evidence gaps, nasal obstruction, rhinosinusitis

## Abstract

Therapeutic decisions in otorhinolaryngology are based on clinical experience, surgical skills, and scientific evidence. Recently, evidence-based therapies have gained increased attention and importance due to their potential to improve the individual patient’s treatment and their potential at the same time to reduce treatment costs. In clinical practice, it is almost impossible to stay ahead of the increasing mass of literature and on the other hand critically assess the presented data. A solid scientific and statistical knowledge as well as a significant amount of spare time are required to detect systematic bias and other errors in study designs, also with respect to assessing whether or not a study should be part of an individual therapeutic decision. Meta-analyses, reviews, and clinical guidelines are, therefore, of increasing importance for evidence-based therapy in clinical practice.

This review is an update of the availability of external evidence for the treatment of nasal obstruction and rhinosinusitis. It becomes evident that both groups of diseases differ significantly in the availability of external evidence. Furthermore, it becomes obvious that surgical treatment options are normally based on evidence of significantly lower quality than medical treatment options.

## General aspects

In this review article, publications focusing on the treatment of nasal obstruction and rhinosinusitis in children are not included because these diseases are very complex and, for example, may encompass congenital diseases. Because it otherwise would have been far beyond the scope of this manuscript, only the evidence and evidence gaps in the treatment of nasal obstruction and rhinosinusitis in adults are described.

## 1 Evidence-based medicine

### 1.1 Basics and short introduction

According to the definition of David Sackett, one of the main protagonists of evidence-based medicine (EbM) is “the conscientious, explicit, and judicious use of current best evidence in making decisions about the care of individual patients” [1]. Specifically, this means a procedure to treat every individual patient based on the best available data. The best available data should be obtained by systematic search and critical assessment. Subsequently, such data should be combined with own clinical expertise and the individual ideas of the patient to make a therapeutic decision. In summary, three pillars are combined: best evidence, individual preferences and needs of the patient, and individual clinical expertise of the treating physician [2]. 

The origins of EbM date back to the middle of the 19^th^ century [1]. Subsequently, the development of EbM was promoted particularly in Canada and Great Britain [3]. The term “evidence based” was first used by Eddy in 1990 [4]. Already at that time, he indicated that in addition to external evidence, the subjective assessment of data is crucial for its application in clinical practice. However, Sackett had already indicated that the flood of publications was unmanageable in parallel with the daily routine. Therefore, in 1996, a general practitioner would already have had to read 19 articles per day to maintain an overview of the entire literature [1].

Since then, EbM has gained significant importance; in Germany, it is included in medical teaching. The German Network for Evidence-Based Medicine (Deutsches Netzwerk für evidenzbasierte Medizin) is actively working on the distribution and development of the methods of EbM. Cochrane Germany is the German partner of the Cochrane Collaboration, an international network (named after the British physician Sir Archibald Leman Cochrane) with the purpose of providing the most reliable information on medical questions. The Association of the Scientific Medical Societies in Germany (Arbeitsgemeinschaft der Wissenschaftlichen Medizinischen Fachgesellschaften e.V., AWMF) coordinates the development of guidelines for diagnostics and therapy by scientific medical societies. The guidelines are based on current scientific knowledge and good practice, and provide an orientation for decision making for clinically active physicians. However, there is the problem in that the contents of guidelines cannot always be put into practice and that contributing to guidelines and systematic review articles is not normally acknowledged at medical faculties [3]. 

Despite such reservations, EbM has the potential to improve prophylaxis as well as the treatment of patients and the outcome and quality of treatment. For example, patients treated according to scientific evidence display higher survival rates [5], better wound healing [6], and shorter durations of inpatient stay [7] compared to patients treated without scientific evidence. Because of this fact, EbM became increasingly important in all medical disciplines in recent years, including otolaryngology.

It is also important to consider that EbM is not limited to randomised trials and meta-analyses, but that in the context of evidence-based therapy decisions, the best available trials are used to make therapeutic decisions [1].

The evidence levels developed by the Oxford Centre for Evidence-Based Medicine (OCEBM) to classify trials according to their value are widely known. Depending on their publication date and the authors’ preferences, most of the trials cited in the present article use the revised version of the OCEBM classification of 2009 (Table 1 (Tab. 1)). Based on the evidence levels, grades of recommendation are defined (Table 2 (Tab. 2)). Subsequently, the OCEBM published a further revision of the 2009 version; Table 3 (Tab. 3) describes this current version of the evidence levels for comparison. Further studies cited here use this current OCEBM classification. In the 2009 version, the grade of evidence of a randomised controlled trial (RCT) is classified as evidence level Ib, in the 2011 version as evidence level 2, which is the best possible grade of evidence for a primary study. Comparisons of study results from trials with historical controls and trials with a randomised design revealed that in studies with historical controls the results were biased through trends in a single direction, which could be avoided by randomisation [8], [9]. RCTs are currently considered the gold standard of study designs [10]. Only meta-analyses and/or systematic review articles that analyse several RCTs and summarise them by using exactly defined methods with a specific question have an even higher value according to the OCEBM – evidence level Ia (2009 version) and evidence level 1 (2011 classification). Categorising randomised controlled trials as having a higher value than observational studies was repeatedly criticised as being too simplified [11], [12]. Currently, there is a consensus that under certain circumstances observational studies [13] and even case reports [14] may additionally provide definitive evidence [11].

In 2000, the international workgroup GRADE (Grading of Recommendations Assessment, Development, and Evaluation) was founded to improve evaluation systems in medicine. The assessment of the quality of evidence and level of recommendation developed by GRADE allows on the one hand enhancement of the value of observational studies, and on the other hand reduces the value of RCTs due to qualitative limitations. Therefore, the above-mentioned disadvantages of too great a simplification of the OCEBM system are avoided. The GRADE system, which is now applied by many organizations, including the WHO, has the further advantage that important factors, including the precision and congruence of results of different studies, are also taken into consideration [11]. Therefore, the GRADE system is more exact than, for example, the OCEBM evidence levels, but it is also clearly more complicated and currently not so widely used compared to the OCEBM classification. Some further studies cited in the present article, particularly the newer Cochrane analyses, use the GRADE system for classification of evidence levels.

In addition to the positive aspects of EbM described above, it has several disadvantages, as stated by Greenhalg and co-workers in 2014 [15] in their paper “evidence-based medicine: a movement in crisis”. In particular, the quality label of EbM was misused by industry through targeted exertion of influence on trials. For example, it was shown that studies of antidepressants sponsored by pharmaceutical companies published a positive outcome in 37 of 38 of the trials, whereas only 14 of 36 studies with negative results were published [16]. Furthermore, it is already impossible to maintain an overview of the large number of guidelines [16]. In addition, the uncritical acceptance of statistically significant differences that only have a low clinical importance could detrimentally effect treatment. Finally, based on the increasingly ageing population and the resulting multi-morbidity, the application of single guidelines is made rather difficult. 

In summary, taking into account the relevant advantages and disadvantages of EbM, it can be stated that its correct application may lead to significant improvements for the patient. However, its application requires critical physicians who consider the individual needs of each patient.

### 1.2 Evidence-based medicine in oto-rhino-laryngology

The absolute number of RCTs among primary studies has increased in all medical disciplines, including otolaryngology [17], [18]. A PubMed search on July 13, 2015, with the key words “evidence based medicine” and “otorhinolaryngology” revealed 764 articles. In the United States of America, EbM in otolaryngology has greatly developed since the start of this century. This fact is reflected in the relatively high number of US-American publications focussed on EbM.

The percentage of prospective trials in the ENT-specific literature is significantly higher than in other disciplines, including the fields of neurosurgery, ophthalmology, and orthopaedics (62 vs. 49%) [19]. A comparison of ENT with general paediatrics, where 6–12% of the published trials of the last 25 years were identified as RCTs, revealed a similar rate of RCTs in both disciplines [17]. Additionally, the number of systematic review articles and meta-analyses published by the Cochrane Library [20] regarding otolaryngology has clearly increased.

In 2006, Wasserman and co-workers analysed how studies and evidence levels in reputable ENT-specific journals developed between 1993 and 2003 [21]. They investigated the original articles of 1993, 1998, and 2003 published in the four most important ENT-specific journals (Annals of Otology, Rhinology, and Laryngology; Archives of Otolaryngology – Head & Neck Surgery; The Laryngoscope; Otolaryngology – Head and Neck Surgery). The authors showed that although the number of publications had increased, most publications still had to be classified as evidence level 4. However, the levels improved slightly with time, with 80% of the therapy studies classified as levels 3–5 and 75% of the diagnostic trials to evidence levels 1 and 2 by 2003.

### 1.3 Challenges in planning and conducting surgical trials

Despite the above-mentioned limitations, randomised controlled trials are still considered the gold standard for confirming the efficacy of new therapeutic procedures [10]. They have the lowest risk for systematic bias [10], [22] and when correctly designed, they control best for placebo effects [22]. However, because of their design, randomised controlled trials can only be used to a limited extent or not at all for surgical procedures. There are significant factors limiting the application of randomised and controlled studies in surgery. On the one hand, there are ethical aspects in cases of so-called sham or placebo interventions. Even when undergoing such placebo interventions, the patient faces the associated surgical risks, including anaesthesia. Depending on the type of surgery, performing such interventions is either not possible or only with important limitations. A comparison with accepted surgical standards is one possibility to avoid such problems. On the other hand, it must be considered that only rarely can all surgical details of an intervention be planned before the surgery itself and that the individual expertise of the surgeon has a high impact on the quality of the outcome [23]. This is certainly the case in complex interventions. However, a retrospective observational study from the Department of Otorhinolaryngology, Head and Neck Surgery, Ulm University Medical Centre demonstrated for otoplasty, which is a less complex intervention, that the results of this intervention on the quality of life and patient satisfaction were independent of the surgeon [24]. Only large multicentre studies can reconcile the above-mentioned differences of complex surgeries. Therefore, such studies can only be conducted for common diseases, whereas experiences with rare diseases have to be reported from single institutions or even individual surgeons, which then represent the best available data and evidence. Furthermore, the surgical tradition of directly transferring knowledge from the teacher to the student makes it difficult to conduct RCTs [22] because the teacher’s knowledge is frequently not questioned. In plastic surgery, randomised controlled trials are relatively rare because of the above-mentioned aspects. In contrast to surgical disciplines, more than 50% of the decisions taken in internal medicine are based on RCTs [25]. 

The focus of sections 2.2.1.1 and 2.2.1.2 will be on the specific challenges in designing surgical studies in the fields of septoplasty and septorhinoplasty. Additionally, potential solutions will be discussed.

### 1.4 Guidelines

Because of the large number of publications that cannot be readily overseen and assessed, one main task of scientific societies is to create guidelines. Within this article, guidelines will also be referred to (*Leitlinie Formstörungen der inneren und äußeren Nase*, Guideline on deformities of the internal and external nose [26]; *Leitlinie Rhinosinusitis*, Guideline on rhinosinusitis [27]). The literature research for this article that was performed on January 5, 2015, with the key words “nasal obstruction” and “treatment”, produced 6,397 hits. Adding “evidence” as a further key word, reduced this to 400 publications, while after the further key word “Cochrane”, 29 manuscripts remained. After only six months, the number of publications had increased to 6,583, 424, and 32, respectively. Such numbers clearly show that it is virtually impossible for the individual physician to maintain an overview of the entire current literature of his/her discipline. Furthermore, the assessment of the quality of each study requires time to analyse adequately the study results in addition to the specific knowledge related to the planning and designing of the trial. Guidelines taking into account the current literature are, thus, highly important for evidence-based therapies.

## 2 Types and therapies of nasal obstruction

### 2.1 Types of acute nasal obstruction

In most of cases, the origin of acute nasal obstruction is an infection. Often, it occurs as an unspecific respiratory infection, as in the case of the common cold. It is necessary to distinguish this from acute nasal obstruction observed in the context of allergic rhinitis. However, initially they often cannot be delineated with certainty by differential diagnosis. Furthermore, specific rhinitis, drug-induced rhinitis, and pregnancy rhinitis may lead to acute nasal obstruction. In addition, acute nasal obstruction may occur after trauma, for example, as sequela of nasal septum fracture, septal haematoma, and septal abscess. In these cases, patient history is essential.

Initially, it is not readily possible to differentiate between acute rhinitis and acute rhinosinusitis based on the clinical symptoms. Because of this fact, the European position paper on the treatment of rhinosinusitis and nasal polyps (EPOS 2012) classifies acute rhinitis as viral rhinosinusitis and thus a subtype of acute rhinosinusitis [28]. The AWMF guideline 017-049 on rhinosinusitis (mainly established by the German ENT Society) [27] adopted the definitions of the European Position Paper of 2012, although acute rhinitis is not included in this guideline. In contrast, the AWMF guideline 053-012 on rhinosinusitis (produced by the German Society of General Medicine) [29] does include acute rhinitis. However, this guideline has not been actualised since 2013. Viral rhinosinusitis, also termed acute rhinitis or the common cold, is characterised by a shorter disease duration (up to a maximum of 10 days) in contrast to acute postviral rhinosinusitis, which lasts for more than 10 days. Acute rhinitis is self-limiting [30]. Both acute rhinitis and acute postviral rhinosinusitis are characterised by a sudden disease onset; at least two of the following symptoms occur: nasal obstruction or anterior or postnasal secretion, facial pain, pressure sensation, and/or loss of olfaction. In the following, only the term “acute rhinitis” will be used to avoid confusion.

In approximately 24–52% of clinical infections, acute rhinitis is caused by rhinoviruses [31], [32], [33], whereas in 31–57% of the infections no pathogen can be detected [33], [34]. In only 5% of the cases can bacteria be confirmed [34].

Due to the incidence and the associated socio-economic relevance, acute rhinitis and its treatment is highly relevant in the clinical practice and daily routine. The following sections will focus only on the therapy of acute rhinitis.

Further causes of acute nasal obstruction, including allergic rhinitis, will not be discussed in this context.

#### 2.1.1 Evidence for different pharmacotherapies

There is a large number of therapeutic options for acute rhinitis, some of which are alternative medicine approaches. The treatment of acute nasal obstruction and acute rhinitis are not included in the current AWMF guidelines despite the common incidence of the diseases (see also 2.1). In contrast, the European Position Paper EPOS 2012 does include the treatment of acute rhinitis as a form of acute rhinosinusitis [28].

##### 2.1.1.1 Decongestants

Several current systematic review articles provide an excellent overview of the different therapeutic options for acute rhinitis [35], [36]. The effectiveness of topical and oral decongestants has been investigated in several trials, with three meta-analyses [37], [38], [39] and one systematic review [40] found.

The meta-analysis of Kollar and co-workers [37], in which seven crossover studies were re-analysed and the data pooled, confirmed the effectiveness of orally applied phenylephrine for the treatment of nasal obstruction in acute rhinitis. However, in a further review, phenylephrine did not have a significant effect on the course of the disease and it remains unclear if longer-lasting effects are present [35]. In summary, the effectiveness must be classified as being uncertain [35].

##### 2.1.1.2 Steroids

In the literature on acute rhinitis treatment, only limited attention is paid to topical steroids, whereas their application in allergic rhinitis [41] and acute postviral rhinosinusitis is well established (see 3.1.1.4). In a controlled trial, test subjects were infected experimentally using rhinoviruses. Before and after exposure, beclomethasone was applied topically. Reduced inflammation was observed within the first two days of experimental infection [42]. In summary, there is neither a sufficient number of trials nor current recommendations that support the application of topical steroids for acute infectious rhinitis because their efficacy for neutrophil inflammation (acute rhinitis) is not proven in contrast to eosinophilic inflammation (allergic rhinitis, nasal polyposis) [43].

##### 2.1.1.3 Antihistamines

An earlier Cochrane analysis from 2003 [44] and an earlier meta-analysis from 1998 [45] as well as a more recent analysis of 22 RCTs [36] showed only a slight improvement of nasal secretion while nasal obstruction persisted after the use of antihistamines alone. A more recent Cochrane analysis from 2012 [46] concluded that antihistamines combined with decongestants and analgesics could reduce the duration of the disease and relieve the symptoms. The authors, therefore, recommended a combination of antihistamines with decongestants and analgesics for adults after considering the possible side effects.

##### 2.1.1.4 Saline solution

Rinsing with saline solution and saline sprays is often part of clinical recommendations for the treatment of acute rhinitis. While a Cochrane analysis from 2010 drew the conclusion that the RCTs included in the analysis did not justify a statement for a positive effect [47], a current Cochrane analysis from 2015 [48] that analysed five RCTs concluded that there is an indication for efficacy of rinsing with saline solution. In a larger RCT with 401 children [49], a significant reduction of nasal secretion and improvement of nasal breathing were found. The group that rinsed their noses with saline solution clearly required less decongestant. In summary, although the authors of the current Cochrane analysis state that saline solution may relieve the symptoms of acute rhinitis, further well-designed RCTs are required for a more exact assessment [48].

##### 2.1.1.5 Vitamin C and zinc

A Cochrane analysis from 2013 assessed the effect of vitamin C on prevention, duration, and severity of acute rhinitis symptoms [50]. In this analysis, exclusively placebo-controlled trials with a total of 11,306 participants were included. In acute rhinitis, a positive preventative effect of vitamin C was observed in subjects exposed to physical strain, whereas this effect was not be confirmed in the normal population. Some of the studies included in this analysis showed a reduction in the severity and duration of acute rhinitis in 3–12% of adults. In the context of the very few therapeutic trials on vitamin C, a preventative effect to date was not confirmed. The authors concluded that due to the positive effect of the prevention trial combined with the very low side effects, vitamin C application could be considered for acute rhinitis [50].

The effect of zinc on the incidence, severity, and duration of acute rhinitis was analysed in another Cochrane review from 2013 [51]. In this investigation, 16 therapeutic and 2 preventive studies were included with 1,387 and 394 participants, respectively. Based on this study, a significant reduction in the duration of the disease was demonstrated, whereas the severity of the symptoms remained unchanged. In summary, the authors recommended application of zinc within 24 hours after disease onset However, they also stated that the data of the studies were relatively heterogenic and that possible side effects, including dysgeusia and nausea, have to be borne in mind [51].

##### 2.1.1.6 Ipratropium bromide

A 2013 Cochrane review analysed the effect of ipratropium bromide compared to placebo and included seven trials that encompassed a total of 1,959 patients (including pediatric patients) with acute rhinitis. While ipratropium bromide had a positive effect on nasal secretion, nasal obstruction was not significantly improved. Because only a few side effects exist, all self-limiting [52], a recommendation for the application of ipratropium bromide was given 

##### 2.1.1.7 Probiotics

Probiotics are preparations containing living microorganisms that are intended to strengthen the immune system. A Cochrane review from 2011 recommended the application of probiotics for the prevention of acute airway infections. In this meta-analysis, data from 10 RCTs with a total of 3,451 participants were included. It concluded that the application of probiotics reduces the number of airway infections, as does the intake of antibiotics, compared to placebo [53].

### 2.2 Types of chronic nasal obstruction

Chronic nasal obstruction may be caused by the underlying anatomy and inflammatory processes, and the origins are manifold. In addition to allergic rhinitis, which is not presented in the present article, and chronic rhinosinusitis (CRS), which is discussed in chapter 3.2, functional nasal obstructions caused by special anatomical variations are the focus of ENT-specific therapy. Hereby, the nasal flow is mainly determined by the shape of the nasal entrance, the nasal cavity, and the turbinates [26]. Because the narrowest point of the nasal airway, the nasal valve, is located in the area of the nasal entrance, deformities in this area play a crucial role. Septal luxation and subluxation, and deviations of the caudal septum end must be considered as well as widening of the columella base, a drooping nasal tip, and deformities of the nasal alae. In the nasal cavity, the deviation of the nasal septum from the median may lead to obstructed nasal breathing. Depending on the location of the deviation within the nose, it may lead to a more or less significant impairment of nasal breathing. Additionally, septal perforations may negatively influence the nasal flow. A pathological shape and size of the turbinates can also lead to nasal obstruction, while the condition of the mucosa, for example, in cases of mucosal hypertrophy, contributes decisively to the function or malfunction of the nasal conchae. In the following, therapy of nasal obstruction caused by anatomical variations will be discussed.

#### 2.2.1 Evidence for surgical therapies

Because of the multitude of specific anatomical situations described briefly above, surgical therapy cannot be as readily standardised, as is the case in pharmacotherapy. Surgical therapy must be adapted to the individual situation of the patient. It is crucial in choosing the appropriate surgical option to accurately analyse the underlying deformity and nasal pathology. However, even with an exact analysis of the pathology, not every ENT specialist will perform the identical or at least a comparable surgery [54], [55] because surgical training and the application of surgical techniques varies enormously not only within Germany but also across Europe and the world. Moreover, there is a multitude of surgical techniques to correct specific deformities, which makes it even more difficult to compare the available procedures. Generally, within the whole discipline of rhinosurgery, more than 85% of the studies are classified as levels IV and V evidence [56]. Studies of higher quality deal mainly with the application of pharmaceutics in the context of rhinoplasty [56].

##### 2.2.1.1 Septoplasty

Septoplasty is one of the most frequently performed ENT-specific interventions. It is performed on both an outpatient and day-clinic basis and under inpatient conditions. During recent decades, the previously common submucous resection of the septum was replaced by septoplasty according to Cottle [57]. Nonetheless, each surgical step of septoplasty according to Cottle varies depending on the individual pathology. A review article published by Moore and Eccles in 2011 [58] analysed 14 trials with 536 patients who had undergone septoplasty. The authors concluded that septoplasty most probably improves objective parameters as determined by rhinomanometry or acoustic rhinometry. They demonstrated that an improvement of the nasal flow by septoplasty had a positive impact on the affected patient. On the other hand, Andre et al. stated in 2009 [59] that a correlation between objectively determined values of rhinomanometry or acoustic rhinometry and subjective complaints of the patients was not clearly proven. However, the correlation was clearer when subjective nasal obstruction was reported. This restriction must be considered when drawing conclusions from the article of Moore and Eccles [58].

In 2004, Row-Jones analysed the existing problems in the design of surgical studies on septoplasty for the treatment of nasal obstruction [60]. A crucial and relevant problem for conducting such trials is that there is no acknowledged classification of septal deviations; only proposals by different authors exist that are not generally acknowledged or have no consensus. Even today, more than 10 years after Row-Jones’ statement, there is no general systematic classification. However, ever more authors [61], [62], [63] have considered these problems, such that a series of reasonable classifications is now available. Recently, Lin et al. suggested a detailed classification based on computed tomographic (CT) data sets [64]. The next step in being able to perform high-quality studies in the field of nasal obstruction and septoplasty would be to establish an international consensus to use one of these classification systems or an appropriate modification thereof for conducting further trials.

In addition to the lack of a standardised classification, Row-Jones cited the lack of general and recognised outcome parameters [60]. In particular, objective measurement methods, including rhinometry and rhinomanometry, are not completely compatible to the subjective symptoms of the patients [59], [65]. On the other hand, Pirilä and Tikanto demonstrated in 2009, that acoustic rhinometry and rhinomanometry may better predict the postoperative satisfaction of patients with less significant septal deviations compared to anterior rhinoscopy [66]. Furthermore, Stewart and co-workers in 2004 already suggested and validated the so-called “NOSE” (*n*asal *o*bstruction *s*ymptom *e*valuation) questionnaire on the quality of life after septoplasty [67], which has frequently been applied and has become acknowledged as a reliable instrument for assessing the subjective satisfaction after septoplasty [68]. In summary, there are presently very appropriate outcome parameters that could be analysed in studies on septoplasty.

Currently, there are several studies of evidence level III available that indicate the effectiveness of septoplasty for the treatment of nasal obstruction [69], when specific pathologies are present.

In summary, once a classification system for septal deviations is agreed upo, it will be clearly possible to design surgical trials with better comparability and transparency and thus generate data with a better level of evidence than currently available studies in the field of septoplasty, being comparable to the area of turbinate surgery.

##### 2.2.1.2 Septorhinoplasty

Septorhinoplasty encompassing septoplasty with correction of the bony nasal pyramid is required in those cases where relevant deformities of the outer nose are present [26].

At present, the number of articles on septorhinoplasty alone can no longer be managed; for example, a PubMed search with the key word “rhinoplasty” performed on July 20, 2015, achieved 7,921 hits. Additionally, in 2015, Leyrer analysed the distribution of articles on rhinoplasty between 1995 and 2011 [56] with regard to the evidence level. Of the 759 articles that were selected for this analysis, 30% were classified as evidence level V, 55.3% as evidence level IV, 5.4% as evidence level III, 4.1% as evidence level II, and 5% as evidence level I. In the course of time, the articles that could be assigned to a higher evidence level increased in the subcategory of “functional aesthetic rhinoplasty”, while most of the studies with evidence levels I and II referred to the administration of pharmaceutics in the context of septorhinoplasty and not to surgical techniques themselves.

Generally, an open (incision in the columella with folding back of the skin and soft tissue mantle) and a closed (hemitransfixion or transfixion incision in the area of the caudal septum end) approach is available. The choice of the approach is based on different factors. Certain problems of the outer nose can be exposed and identified more clearly by an open approach [55], which is particularly important when revision surgery, intervention on the nasal tip, or surgery of cleft nose is indicated. In endonasal rhinoplasty, it is beneficial to avoid a scar in the columella [70] and tissue trauma is much less than with open an approach. On the other hand, visibility and identification of underlying anatomy and pathology is poorer in comparison to the open approach [71], making surgery of complex anatomical deformities more difficult to perform [55]. Independent of the advantages and disadvantages of the approaches, rhino-surgeons often make their decision for an open or closed approach depending on their individual surgical expertise [54], [55]. Currently, there is only one study comparing an open to a closed approach in the field of correction of cleft noses: In 2002, Rettinger and O’Connel compared the surgical outcome of using a closed approach with an open approach including the application of composite grafts in a retrospective analysis of 54 patients with a follow-up period of up to 42 months [72]. More stable results were observed with regard to breathing function and symmetry after an open approach and the application of composite grafts. Even though this study was a retrospective analysis, the results remain – even after more than 10 years – the best available data with regard to long-term results of different reconstruction techniques of cleft nose deformities. Further trials comparing different approaches for the treatment of fractures of the nasal pyramid have been published. Reilly and co-workers compared, for example, a classical closed fracture reposition with rhinoplasty techniques [73], while another study prospectively compared an open approach to the nasal septum with the closed reposition for treatment of fractures of the nasal pyramid [74]. In both studies, classified as evidence level III, the open approaches appear to allow a better exposition of the pathology and to be beneficial with regard to the probability of revision and recurrent deformity.

In 2011, Lee and co-workers in a systematic review article summarised the current literature on the treatment of the nasal dorsum. Then as now, no comparative studies were identified that compare, for example, a sequential reduction with an en-bloc-reduction of the nasal dorsum [75]. While different retrospective case series describe the applied technique and the outcome, comparisons of different techniques are not possible. Although there is almost a consensus regarding augmentation of the nasal dorsum that autologous cartilage is the gold standard, there remains a considerable difference of opinion regarding the donor site and further treatment of the inserted cartilage [75]. Additionally, irradiated costal cartilage and bone transplants appear to be acceptable materials for augmentation of the nasal dorsum. The use of synthetic implants is controversial; in particular, the application of silicone is considered very critically [75] or fully objected [76] because of potential risks, including extrusion and skin necrosis. Even when the use of Gore-Tex appears to be less risky, the indication should be made very carefully because, there is also a risk of extrusion [75].

Many other aspects of rhinoplasty, including reconstruction of saddle noses [77] and surgery of the nasal valve, nasal tip, and middle third of the nose, are currently not analysed in comparative prospective studies, although this is urgently required for the future development of rhinosurgery [75].

Therefore, while there is a general lack of studies comparing different rhinosurgical techniques, a series of trials have now been performed that analysed important aspects of rhinosurgery, including the quality of life after septorhinoplasty and the necessity of antibiotics or steroids. Several authors demonstrated that septorhinoplasty significantly improved the quality of life of affected patients [78]. However, in most of cases, these are retrospective studies [79], the significance of which is limited because of methodical shortcomings.

A Cochrane analysis on the effect of perioperative steroids in preventing complications after plastic facial surgery was published in 2014 [80]. In this analysis, 10 randomised controlled trials with a total of 422 patients were included, with nine rhinoplasty studies and one facelift study. These studies investigated the effect of steroids on the development of haematoma and postoperative swelling. In summary, the Cochrane analysis revealed a reduction in swelling and haematoma within the first two postoperative days after application of a single 10 mg dose of methylprednisolone. Only in one trial, where 250 mg of methylprednisolone were applied, was it was concluded that this application could reduce oedema and haematoma between the first and seventh postoperative days. In summary, the available studies were in part contradictory; complications were analysed only to a limited extent so that a recommendation for the application of steroids to reduce perioperative complications of rhinoplasty could only be given with reservation. Therefore, also in this field, are further studies needed. A recently published randomised and double-blind study of 42 patients demonstrated that preoperative intravenous application of dexamethasone reduced oedema and haematoma for 7 days after surgery [81]. Therefore, the data available on the application of dexamethasone has improved since the Cochrane review. A recent meta-analysis from 2015 concluded that steroids are appropriate to reduce postoperative oedema and haematoma development after septorhinoplasty [82].

Studies on perioperative application of antibiotics for plastic surgery, including seven studies on septorhinoplasty and septoplasty, were recently included in a meta-analysis of an American expert group [83]. The authors concluded that for interventions at contaminated areas of the head and neck, including septoplasty and septorhinoplasty, perioperative antibiotic therapy can be recommended (to a limited degree) even though the available randomised trials were associated with a high risk of systematic failure. Long-term antibiotic therapy is not recommended [83].

##### 2.2.1.3 Turbinate surgery

Hypertrophy of the nasal turbinates is another common cause of chronic nasal obstruction [84]. The aetiology of hypertrophy of nasal turbinates is variable, including amongst others allergic rhinitis, non-allergic rhinitis, chronic hypertrophic rhinitis, and compensatory turbinate hypertrophy. If pharmacotherapy with, for example, decongestants, steroids, or antihistamines does not improve the symptoms, another therapeutic option is the surgical reduction of the turbinates. Currently, it remains unclarified when the indication for surgery of the turbinates should be made.

Regarding the surgical reduction of nasal turbinates, there is a large number of different surgical techniques, encompassing conventional reduction of the inferior turbinates anterior turbinoplasty, partial or total turbinoplasty up to laser-assisted techniques, cryosurgery, microdebridement, and ultrasound-guided reduction of the inferior turbinates. Currently, there is no broad consensus as to which procedure provides the best long-term results, although five randomised controlled trials of evidence level I were published between 2003 and 2010 that considered different techniques of reduction of nasal turbinates. In 2004, Nease and Krempl [85] compared the effectiveness of radiofrequency ablation of the inferior turbinates in a prospective and randomised study with placebo treatment consisting of insertion of the radiofrequency probe without energy application. Based on subjective visual analogue scales, they demonstrated that the active treatment led to significantly improved nasal breathing after 6 months in comparison with placebo. In 2003, Passali and co-workers performed a comparative analysis of six different techniques (turbinectomy, laser therapy, electrocautery, cryotherapy, submucous resection, and submucous resection with lateral fracture) for the reduction of the inferior turbinates with a follow-up period of up to 6 years [86]. In this study, the authors showed that the submucous resection with lateral fracture provided the best long-term outcome with regard to nasal breathing. In contrast, Liu and co-workers, analysed 120 patients by considering visual analogue scales and rhinomanometry, and found that microdebrider-assisted reduction of the inferior turbinates was more effective up to three years after treatment than radiofrequency therapy [87]. In 2010, Cingi et al. [88] published similar results, also in a prospective study, with 268 patients that confirmed the data of Liu et al. However, both these studies did not perform randomisation of the patients, and Cingi and co-workers apparently excluded treatment failures (no improvement of the symptoms after treatment) from the analysis, clearly limiting the significance of this study. In summary, in nasal turbinate surgery, a significantly greater number of high-quality trials exists in comparison to septoplasty and septorhinoplasty. This fact can be best explained by the comparably simple surgical techniques and the possibility to detect intra-individual differences in individual patients.

**Figure 1:**
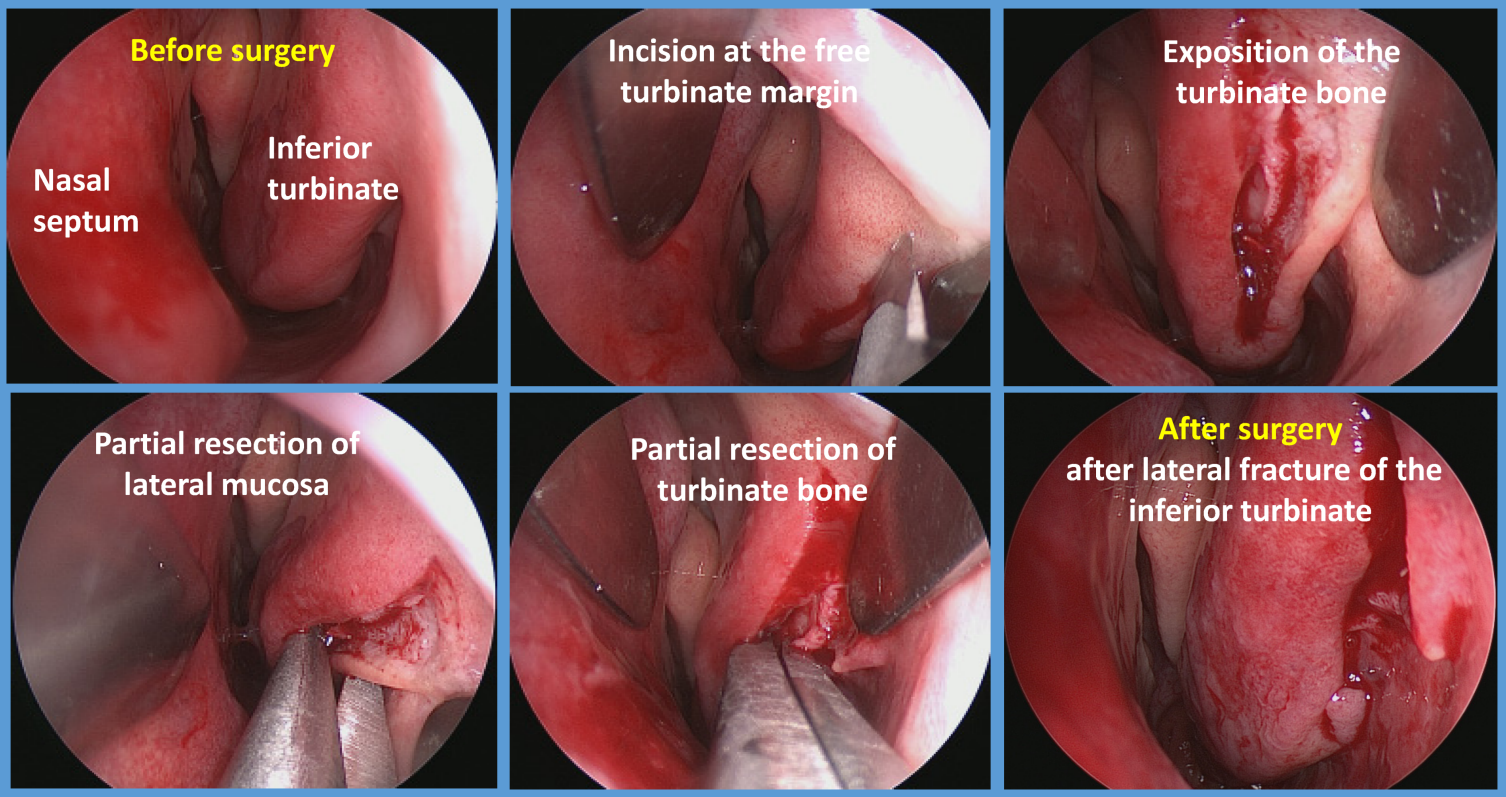
Submucous resection with lateral fracture (anterior turbinoplasty): surgical technique

In summary, regarding conventional techniques, submucous resection with lateral fracture (Figure 1 (Fig. 1)) and microdebrider treatment of the inferior turbinates can be recommended. Because the trials compared different techniques, direct comparison of both procedures is currently not possible.

### 2.3 Evidence for non-surgical and non-pharmaceutical therapies

In addition to classical surgical procedures for the improvement of nasal breathing, further therapeutic options exist, particularly for the treatment of too narrow nasal valves, including minimally invasive procedures on the one hand and widening nasal strips on the other hand [89], [90], [91], [92]. However, for both therapeutic options, there are very few scientific publications. These publications are classified as evidence level V and currently do not allow appropriate integration of the conclusions in the clinical routine.

## 3 Types and therapies of rhinosinusitis

### 3.1 Types of acute rhinosinusitis

Acute rhinosinusitis is characterised by a sudden onset of the disease associated with nasal obstruction or anterior or postnasal secretion and/or facial pain and/or sense of pressure and/or loss of olfaction. Acute rhinosinusitis is subdivided into viral, post-viral, and acute bacterial rhinosinusitis [28]. Viral rhinosinusitis is synonymous to acute (viral) rhinitis and has a maximum duration of 10 days. In post-viral rhinosinusitis, intensity of the symptoms increases after 5 days or symptoms last longer than 10 days, with a maximum of 12 weeks. Acute bacterial rhinosinusitis occurs in only a small percentage of the patients and is characterised by purulent secretion from the nose, severe local pains, fever, increased c-reactive protein (CRP), and a biphasic course with deterioration after an initially mild course [28]. It similarly persists for a maximum of 12 weeks (Table 4 (Tab. 4)).

#### 3.1.1 Evidence for pharmacotherapies

In comparison to the treatment of nasal obstruction, the treatment of rhinosinusitis is much better investigated. There are numerous publications with high evidence levels and current guidelines focusing on rhinosinusitis. The current guideline on rhinosinusitis of the German Society of Oto-Rhino-Laryngology, Head & Neck Surgery dates from March 2011 and was valid until the end of February 2016 [27], [30]. Furthermore, the EPOS from 2012 [28] is particularly applied. This is a revised version of the position papers of 2005 and 2007. Generally, acute rhinosinusitis must be distinguished from chronic rhinosinusitis with and without nasal polyps. Furthermore, the EPOS defines a type of rhinosinusitis that is difficult to treat and is observed in patients who do not respond satisfactorily to maximal pharmacotherapy or surgical therapy to control the symptoms.

##### 3.1.1.1 Antibiotics

A series of meta-analysis of RCTs showed that acute rhinosinusitis heals without antibiotic treatment in approximately 80% of cases [93], [94], [95] and that symptomatic therapy is sufficient. In contrast, the application of antibiotics only slightly increases healing (90%) and must be discussed carefully with regard to their side effects [95]. Therefore, antibiotic therapy should be restricted to cases of bacterial rhinosinusitis that are associated with high fever and severe pains. The first line of therapy should then be antibiotics with a narrow spectrum, including penicillin and amoxicillin, which treat the most frequently occurring pathogens (Streptococcus pneumoniae and Haemophilus influenzae) [96]. Generally, short-term antibiotic therapy (mean duration of 5 days up to a maximum of 7 days) should be preferred to long-term application over 10 days to reduce the occurrence of side effects, increase compliance, lower the frequency of resistance development, and at the same time lower the costs [97]. Even though Germany is one of the European countries with a relatively low antibiotics consumption [98], it must still be considered that the resistance rate is directly correlated to the consumption rate [99], thus unnecessary application of antibiotics should be avoided.

##### 3.1.1.2 Topical steroids

The prescription of topical steroids was already recommended in the EPOS of 2007 [100] as a monotherapy for the treatment of mild acute rhinosinusitis and in combination with antibiotics for the treatment of acute bacterial rhinosinusitis. This recommendation was based on a series of RCTs [101], [102], [103], [104] and a Cochrane Review from 2009 [105]. Additionally, the current guideline of the German Society of Oto-Rhino-Laryngology, Head and Neck Surgery recommends the application of topical steroids. It is interesting to note that a randomised controlled multicentre trial involving 981 patients by Meltzer from 2005 [101] demonstrated the greater efficacy of the application of two shots of mometasone spray over 15 days compared to placebo and compared to antibiotic therapy with amoxicillin for 10 days. The authors reported that the mean symptom score (sum of the scores for rhinorrhoea, postnasal secretion, nasal obstruction, headaches, and facial pressure sensation) was significantly lower after the treatment with two shots of mometasone spray than after treatment with amoxicillin or placebo. An increased recurrence rate or the increased occurrence of bacterial infections was not observed up to 14 days after the end of medication in this study [101].

##### 3.1.1.3 Systemic steroids

A current Cochrane review from 2014 promotes the symptomatic effectiveness (improvement of headaches, facial pains, and nasal obstruction) of systemic steroids combined with antibiotic therapy for acute rhinosinusitis while the administration of systemic steroids alone cannot be recommended [106]. However, the trials included had the risk of systematic failure, such that high-quality studies should be performed with regard to therapy with systemic steroids, particularly taking into consideration the profile of side effects of this therapy.

##### 3.1.1.4 Decongestants

Decongesting nose drops are frequently used for therapy of acute rhinosinusitis. However, there is only a low-level evidence for their efficacy. Although an improved mucociliary clearance was observed, there was no effect on the general course of the disease [107]. Even the combination with antibiotic agents did not positively influence headaches or nasal obstruction [108]. Only in acute viral rhinosinusitis was a positive effect on nasal obstruction observed after 3–10 hours [35]. In a Cochrane review [46], only the combination of decongestants with antihistamines and analgesics were shown to reduce the disease duration and relieve the symptoms, such that these combinations were recommended as a treatment for adults when the side effects are carefully taken into consideration.

##### 3.1.1.5 Antihistamines

Antihistamines represent the standard treatment for allergic rhinitis [109]. They are also often prescribed for acute rhinosinusitis [28], [110]. However, antihistamines are only recommended for the treatment of acute rhinosinusitis when allergic rhinitis is concurrently present [28].

##### 3.1.1.6 Saline solution

Local rinsing of the nose with isotonic or hypertonic saline solution is part of several clinical recommendations. Regarding its effectiveness, this is currently considered as being rather low, with only limited evidence provided. A current Cochrane review from 2015 [48] summarised some positive aspects of rinsing with saline solution for the treatment of acute rhinosinusitis. However, insufficient study designs were indicated as a relevant limitation. One trial reported a lower absence from work while a larger trial encompassing pediatric patients revealed a reduction of nasal obstruction and nasal secretion [49] (see also 2.1.1.4). Another meta-analysis showed a minor positive effect of nasal rinsing with saline solution [111]. Therefore, because no relevant side effects are reported, this treatment can be recommended as a supportive measure.

Inhalations of hot steam may relieve the symptoms of acute viral rhinosinusitis (acute rhinitis). However, the Cochrane analysis revealed no consistent positive effect [112]. Therefore, that the application of inhalations is not recommended in the current EPOS [28].

##### 3.1.1.7 Secretolytic and herbal agents

Secretolytic agents are not recommended either in the guideline on rhinosinusitis [27] or in the EPOS [28], even though they are frequently used in daily practice. There is more evidence for the effectiveness of herbal agents, for example, standardized myrtol, which significantly improved the symptoms of acute rhinosinusitis in a placebo-controlled study in comparison to placebo [113]. In addition, a recent prospective, placebo-controlled study with 386 patients demonstrated the effectiveness of phytopharmaceuticals with several components in comparison with placebo [114]. Another open trial recently confirmed these results [115]. 

### 3.2 Types of chronic rhinosinusitis

In the literature, the definition of chronic rhinosinusitis is inconsistent and encompasses a large number of different disease subtypes. The following sections will focus on definitions of the EPOS [28] and the guidelines on rhinosinusitis of the German Society of Oto-Rhino-Laryngology, Head and Neck Surgery [27] (Table 5 (Tab. 5)). In contrast to acute rhinosinusitis, chronic rhinosinusitis is characterised by symptoms persisting for more than 12 weeks. The disease is defined as an inflammation of the nasal cavity and adjacent paranasal sinuses. In addition to nasal obstruction or nasal secretion, at least one of the following symptoms must also be diagnosed:

- Facial pains or pressure sensation
- Reduced olfaction or loss of olfactory function

Furthermore, endoscopy must demonstrate one of the following aspects:

- Polyps in the middle meatus and/or
- Purulent secretion in the middle meatus and/or
- Mucosal oedema in the middle meatus.

Additionally or alternatively, opacity may be observed in the CT scan of the paranasal sinuses.

In addition to local symptoms, further symptoms, including ear- and toothache, sore throat, dysphonia, and a cough as well as a general feeling of illness and fever are frequently observed [28]. Overall, the symptoms of chronic rhinosinusitis are milder compared to acute rhinosinusitis.

The European Position Paper of 2012 stringently differentiates between chronic rhinosinusitis with and without nasal polyps, because these diseases are clearly different with regard to the inflammation and subsequently the therapeutic outcome. In cases of chronic rhinosinusitis with polyps, the above-mentioned symptoms are accompanied by nasal polyps, which are endoscopically visible in the middle meatus. In contrast, no polyps can be identified endoscopically in the middle meatus in chronic rhinosinusitis without polyps.

Furthermore, the clinical experience justifies the differentiation between chronic rhinosinusitis with and without polyps. However, in terms of evidence, there is the problem that most current studies do not differentiate between both entities and that the outcome is frequently described for both diseases together.

Another type of chronic sinusitis with nasal polyps is present when the patient also suffers from asthma and additionally from aspirin or other nonsteroidal anti-inflammatory drugs (NSAID) hypersensitivity. Such a condition is termed “acetyl salicylic acid intolerance syndrome” [116] or AERD (aspirin exacerbated respiratory disease) [28], formerly known as Samter’s triad. This disease is characterised by a particularly high tendency of recurrence and the need for revision surgery [117], [118].

#### 3.2.1 Chronic rhinosinusitis without nasal polyps – evidence for pharmacotherapies

##### 3.2.1.1 Topical steroids

Topical steroids are recommended based on high-quality trials with evidence level Ia for the treatment of chronic rhinosinusitis with and without polyps (grade of recommendation A). The EPOS indicates 11 studies that clearly confirmed the efficacy [119], [120], [121], [122], [123], [124], [125], [126], [127]. One study compared different application forms of steroids [127], while another compared the effect of topical steroids with antibiotics to antibiotic treatment alone [128]. Nine further studies investigated topical steroids versus placebo. A meta-analysis of five of these trials was conducted, confirming a significant advantage of applying topical steroids. Only one of the trials failed to confirm a positive effect of topical steroids [120]. Comparing patients who had already undergone surgery with those who had not been operated, the effect of topical steroids becomes even more obvious in the sense that patients benefited more after surgery. The analyses published to date do not show significant differences between modern and older topical steroids, although modern steroids have less side effects. The side effects of topical steroids include epistaxis, dry mucosa, and burning sensations. The patients concerned usually tolerate those side effects well.

##### 3.2.1.2 Systemic steroids

In contrast to topical steroids, the effect of systemic steroids in the treatment of CRS without nasal polyps is less well demonstrated. Lai and co-workers identified 27 studies on this topic for an analysis published in 2011 [129]. However, only one of these studies had a prospective design, there were no RCTs, and all the studies applied oral steroids combined with topical steroids and antibiotics. Only three of these studies displayed a positive effect of systemic steroids [130], [131], [132]. Because of the side effects of systemic steroids, only a weak recommendation is given for the application of systemic steroids (grade of recommendation C). 

##### 3.2.1.3 Antibiotics

**Short-term application**

Regarding the short-term application (up to 4 weeks) of antibiotics, there are no placebo-controlled trials. Trials have only been conducted that compared different antibiotics for the treatment of acute exacerbation of chronic rhinosinusitis. No significant differences were found between the antibiotics with regard to their effectiveness [133], [134], [135]. In 206 patients, amoxicillin/clavulanic acid and cefuroxime were compared and a similar response rate was observed. However, a clearly higher recurrence rate was found after cefuroxime application in comparison to amoxicillin/clavulanic acid. In another study with 251 patients, ciprofloxacin and amoxicillin/clavulanic acid were compared. Here, a comparable response rate was observed initially. However, after 40 days, significantly more patients from the ciprofloxacin group were healed, whereas gastrointestinal side effects were observed more frequently in the amoxicillin/clavulanic acid group. Therefore, antibiotics are recommended for short-term application only in cases of acute exacerbations (recommendation grade B).

**Long-term therapy**

The long-term application of macrolide antibiotics is the topic of numerous investigations. Macrolide antibiotics have been applied successfully in pulmonology for many years, for example, to treat diffuse panbronchiolitis, even though the evidence is rather poor according to a current Cochrane review article [136], the authors identifying only one eligible RCT with 19 patients. Other studies published with greater numbers of patients [137] show a clear increase of the 10-year survival rate of those patients who were not included in the Cochrane analysis because of inappropriate methods. For the treatment of cystic fibrosis with clarithromycin and azithromycin, there are a series of RCTs and a current Cochrane review that convincingly confirm that inflammation markers decrease with treatment, the rate of exacerbations is reduced, and the deterioration of the lung function is decelerated [138]. Even for therapy of bronchial asthma, it was shown that macrolides reduce inflammation markers and decrease the pulmonary hyperreactivity [139], [140], [141]. However, not all patients suffering from asthma appear to benefit in the same way [142].

In the treatment of chronic rhinosinusitis, the evidence for the long-term application of macrolide antibiotics is poor. To date, only two placebo-controlled trials exist on this topic [143], [144], which reported contradictory results. While one of the studies demonstrated a positive outcome of a 12-week therapy with low-dose roxithromycin (150 mg/d) in patients with chronic rhinosinusitis without nasal polyps, the other trial did not reveal a positive effect of a 12-week application of azithromycin (500 mg/week) in patients with chronic sinusitis with or without nasal polyps. A trial published by Wallwork and co-workers in 2005 [143] revealed a significant improvement of the SNOT-20 score of nasal endoscopy, and of IL-8 levels. A recent review [145] did not identify further RCTs. The conclusion was drawn that particularly patients with chronic rhinosinusitis (CRS) without polyps and normal immunoglobulin E (IgE) values may significantly benefit from therapy with roxithromycin (grade of recommendation A). However, the authors point out that the indication to apply macrolides must be made very carefully for patients with an increased risk of cardiovascular events, because these substances may trigger cardiac arrhythmia. Therefore, regarding the application of macrolides, further studies are urgently needed that include exactly defined subpopulations of patients with chronic rhinosinusitis. Therefore, the long-term application of macrolides can currently be recommended only for patients who have a regular IgE level and who do not respond to topical steroids or rinsing with saline solution.

##### 3.2.1.4 Topical application of anti-infective agents

**Topical antibiotics**

All placebo-controlled studies investigating the effect of topical antibiotics failed to confirm a positive outcome [125], [146], [147]. An earlier trial from 1986 compared the effect of dexamethasone, neomycin, and tramazoline with dexamethasone with placebo. A study from 2001 compared tobramycin saline solution with saline solution alone applied in 20 patients. A trial from 2008 comparing the outcome of nasal rinsing with bacitracin/colimycine with placebo was restricted to 14 patients. In all three publications, the results with and without antibiotics were similar. Only some open trials revealed a positive effect of additional antibiotics. Therefore, the European Position Paper does not recommend the topical application of antibiotics (grade of non-recommendation A). A more recent review article by Lee and Chiu [148] concluded that such rinsings cannot be recommended as standard. However, in cases that do not respond to established therapy, they may be taken into consideration.

**Topical amphotericin B**

In the EPOS and in a recent review article by Wang et al. [149], the recommendation to not use topical amphotericin B is made This is based on the fact, that five RCTs were unable to demonstrate a positive outcome in nasal endoscopy or in the culture of fungi from the nasal mucosa.

##### 3.2.1.5 Saline solution

In contrast to the treatment of acute rhinosinusitis, rinsing with saline solution is recommended in the European Position Paper for the treatment of chronic rhinosinusitis (grade of recommendation A), even though for CRS the data situation does not appear to be much better than for acute rhinosinusitis. A Cochrane review article from 2007 [150] considers nasal rinsing as useful for the relief of the symptoms of chronic rhinosinusitis. However, no difference was found between CRS with and without polyps. An RCT comparing nasal rinsing to nasal spray with saline solution in 127 patients revealed nasal rinsing to be more effective compared to spray [151].

##### 3.2.1.6 Other substances

Probiotics are preparations containing living microorganisms that are intended to stimulate the immune system. The efficacy of probiotics recommended for the prevention of acute rhinosinusitis (2.1.1.7) was not proven for CRS. Regarding this topic, one RCT was found with 77 patients treated with a probiotic preparation of Lactobacillus rhamnosus vs. oral placebo [152]. No difference was identified on the intake of the agents with regard to symptoms or the SNOT-20 score.

An increased association between gastroesophageal reflux and chronic rhinosinusitis was found in the context of epidemiological studies. Proton-pump inhibitors might be therapeutically effective [153] because gastroesophageal reflux is discussed as possible origin of chronic rhinosinusitis [154]. However, to date, it was only shown that the application of proton-pump inhibitors may reduce postnasal secretion [155], whereas an effect on other symptoms of chronic rhinosinusitis was not identified [28].

#### 3.2.2 Chronic rhinosinusitis with nasal polyps – evidence for pharmacotherapies

As indicated above, many trials do not differentiate between CRS with or without nasal polyps and the therapy of CRS without nasal polyps has already been described above as far as it is definable. Therefore, the following paragraphs will focus only on the differences and the specific aspects of chronic rhinosinusitis with nasal polyps that have not been discussed in section 3.2.1.

##### 3.2.2.1 Topical steroids

As described earlier in section 3.2.1.1, topical steroids are also recommended with grade A for the treatment of CRS with nasal polyps. In addition to a significant improvement of nasal obstruction, meta-analyses confirmed a certain degree of polyp reduction [28].

##### 3.2.2.2 Systemic steroids

In contrast to CRS without nasal polyps, the positive effect of systemic steroids for the treatment of CRS with nasal polyps has, in the meantime, been clearly confirmed by several RCTs [156], [157], [158], [159], [160]. The quality of life of the patients and the nasal obstruction is improved and the size of the polyps reduced. The application of systemic steroids is currently recommended with grade A, based on the described and further studies. However, it must be considered that the positive effect of systemic steroids is only temporary and thus repeated administration must be weighed against possible side effects of a systemic steroid therapy.

##### 3.2.2.3 Antibiotics

The application of doxycycline for 20 days was effective in reducing the size of polyps in an RCT by Van Zele and co-workers from 2010. Furthermore, doxycycline reduced postnasal secretion. Similar to the effect on nasal obstruction, rhinorrhoea, and loss of olfaction, this effect was less than that of methylprednisolone [158]. However, the effect lasted for 12 weeks, while the group treated with oral steroids observed an effect for only 8 weeks. More recent studies could not be found. The current grade of recommendation in the EPOS is grade C [100]. Therefore, also in this context, further studies are required. The long-term antibiotic therapy for CRS with nasal polyps has to date been examined to a lesser extent than CRS without nasal polyps. Only one recent randomised controlled trial deals with the effect of clarithromycin for 12 and 24 weeks combined with topical steroids in the postoperative treatment of CRS with nasal polyps after functional endoscopic sinus surgery (FESS) for up to 6 months. In this study, an improvement of the endoscopic and CT scores was reported [161]. In the European Position Paper, only open uncontrolled trials are discussed and the grade of recommendation is currently C, based on level III evidence.

##### 3.2.2.4 Other substances

Anti-IgE and anti-interleukin-5 antibodies are currently not recommended, neither are leukotriene antagonists or antimycotic agents for the therapy of chronic rhinosinusitis with nasal polyps [28].

Because the absolute IgE level is often increased in patients with CRS with nasal polyps, treatment of this disease with the anti-IgE antibody omalizumab was assessed in an RCT by Pinto and co-workers in 2010 [162]. In this study, an improvement of the SNOT-20 score was observed after 3, 5, and 6 months in the omalizumab group. However, overall there were only limited differences compared to the placebo group and thus only a very small positive effect. In contrast, a more recent RCT in patients suffering from CRS with nasal polyps and asthma showed a significant size reduction of the polyps and an improvement in the quality of life [163], such that therapy with omalizumab can be considered for this patient group.

Interleukin-5 is frequently found in the blood, nasal secretion, and polyps of patients suffering from CRS with polyps. However, only limited data is available regarding the application of IL-5 antibodies. In 2005, Gevaert and co-workers showed that the injection of the IL-5 antibody reslizumab might reduce the size of the polyps by 50% [164]. Another IL-5 antibody, mepolizumab, was assessed by the same group in a separate RCT, with 30 patients who did not respond to topical steroids [165]. Therapy with this antibody also significantly reduced the size of the polyps one month after two injections. Therefore, such therapies can be recommended in cases of failure of established therapeutic approaches.

Leukotriene antagonists are currently not approved for CRS therapy [116]. In contrast to the EPOS Position Paper of 2012, a recent meta-analysis that included 12 trials, came to the conclusion that leukotriene antagonists may reduce the size of polyps and significantly improve different symptoms of CRS, including headaches, facial pains, postnasal secretion, and olfactory disorders in comparison to placebo [166]. Because the results varied significantly between the trials, further RCTs are necessary to determine in which combination leukotriene antagonists should be applied and which patients may benefit most from their application.

#### 3.2.3 Aspirin-exacerbated respiratory disease – evidence for pharmacotherapies

##### 3.2.3.1 Aspirin desensitisation 

The therapy of aspirin-exacerbated respiratory disease is generally performed in analogy to therapy of CRS with nasal polyps, primarily with topical and systemic steroids (see 3.2.2.1 and 3.2.2.2). Because the recurrence rates after FESS are very high in aspirin-exacerbated respiratory disease, this therapy must be complemented using special pharmaceutic therapies. Aspirin desensitisation in the meantime is acknowledged as the standard therapy for such patients and is recommended in the guideline on rhinosinusitis [30] and other publications [116], [167]. In this therapy, the application of ASA induces tolerance to NSAIDs [168]. Consequently, the sinonasal and asthmatic symptoms improve, the recurrence rates decrease, and the size of the polyps is reduced [117], [169], which further leads to a reduction of sinus revision surgery. Currently, oral application is considered the therapy of choice [116], [167]. The effect of aspirin desensitisation was convincingly confirmed by several prospective, partly and fully randomised, double-blind studies [117], [170], [171]. Regarding the optimal dosage, there is currently no consensus, because dosages of 100, 300, and 500 mg were all effective [116]. The most frequently applied maintenance dose is 300 mg [116], [117].

The additional application of leukotriene antagonists is only possible with simultaneous bronchial asthma because these substances are only approved for the treatment of bronchial asthma and not for CRS with nasal polyps [116]. In CRS with nasal polyps, the application of leukotriene antagonists significantly improved the quality of life of the affected patients [172].

#### 3.2.4 Chronic rhinosinusitis with and without nasal polyps – evidence for surgical procedures

##### 3.2.4.1 Functional endoscopic sinus surgery

The number of FESS has increased continuously, with, for example, in the USA alone approximately 250,000 interventions performed annually on an outpatient basis [173]. The problem of conducting randomised controlled surgical trials was discussed earlier in section 1.3.

The consensus in the literature is that the indication for FESS is given when pharmacotherapy is unsuccessful [28]. Furthermore, large trials showed that FESS may clearly improve the symptoms of chronic rhinosinusitis (SNOT-22 score) in patients with and without nasal polyps [174]. Several meta-analyses confirmed the safety and efficacy of FESS [175].

Nonetheless, there is no generally accepted procedure when conservative treatment should be replaced by surgical measures [176], [177]. In a Cochrane analysis of 2014 [176], pharmaceutic treatments were compared to FESS. Four prospective randomized trials with 231 patients were included. One of these studies compared FESS to the application of systemic steroids [178], another compared FESS combined with a topical steroid to the application of antibiotics combined with a topical steroid [179], while the two remaining studies compared polypectomy to systemic steroids [180], [181], [182]. In summary, with regard to the indication for a surgical procedure, the surgical procedure itself, and the postoperative treatment, these four trials were very heterogenic, such that the results cannot really be compared. Even the authors of the Cochrane review concluded that the present studies must be considered as limited and that no reasonable conclusions can be drawn. Similarly, other recent studies showing a greater efficacy of the surgical procedure have significant methodical limitations, including a lack of randomisation and unusual treatment regimen. For example, the patients were not treated according to the current standard (i.e., first with pharmaceutics and then surgery) [183].

Currently, it can be stated that functional endoscopic sinus surgery is an effective measure to treat chronic rhinosinusitis. Furthermore, there is consensus that surgery should only be indicated after maximum pharmaceutic therapy [177]. However, when and to what extent surgery is superior to pharmacotherapy remains an open question.

Whether endoscopic surgery achieves similar results as conventional surgery (e.g. Caldwell Luc surgery), was analysed in several retrospective studies [184], [185]. In this particular context, the endoscopic procedure proved more effective. Of course, there are always systematic flaws because of the study design. Moreover, prospective trials on such questions are no longer reasonable or justifiable due to the good study situation with functional endoscopic sinus surgery.

##### 3.2.4.2 Balloon sinuplasty

Only recently were the results of a prospective randomised study presented that included 135 patients who underwent either conventional FESS or balloon dilatation [186]. This study compared the results of the different procedures after 6 and 24 months, the authors concluding that both procedures achieved similar results. However, balloon dilatation was characterised by less postoperative pains and more rapid recovery from the intervention. Furthermore, the authors performed a meta-analysis, showing that the treatment of 358 patients by balloon dilatation led to satisfactory results. An earlier Cochrane analysis performed in 2011 concluded that the significance of balloon dilatation in comparison to FESS was not clearly defined [187], and thus further studies were urgently required. However, in Germany, after an initial peak phase of application of balloon sinuplasty, its use clearly appears to be declining, due to a rather ambiguous data situation.

##### 3.2.4.3 Microdebrider/powered instruments for FESS

The application of the microdebrider was introduced in paranasal sinus surgery by Setliff in 1994. Subsequently, some studies compared the use of this instrument to conventional techniques. Grevers applied such a system in 1995 for the first time and advocated its use in particular for the surgery of nasal polyps because in addition to providing an almost bloodless intraoperative situs, it allows exact cutting of structures, whereas conventional techniques were considered as being less exact [188]. In 1996, Krouse and Christmas also favoured the application of a microdebrider technique in a non-prospective, non-randomised trial with 250 patients, because its use resulted in less intraoperative bleeding and a lower number of postoperative synechia as well as more rapid healing [189].

In contrast, a prospective randomised study conducted by Sauer in 2007 revealed that the application of powered instruments offered no advantages with regard to endoscopic postoperative results after 6 months, with a slight advantage observed only 3 weeks after the intervention in the group treated with powered instruments [190]. Recently, Singh and co-workers [191] found no advantage with regard to intraoperative bleeding and the duration of surgery. Therefore, the application of a microdebrider for surgery of the paranasal sinuses currently remains without any evidence for the advantages of the application of this system.

##### 3.2.4.4 Computer-aided surgery

The use of navigation systems is increasing in functional endoscopic sinus surgery. Its objective is the improvement of intraoperative orientation [192], [193], particularly in cases of anatomical variations (e.g. after previous surgeries). For example, Jiang et al. recently showed in a retrospective study that opening of the sphenoid sinus in revision surgery is performed significantly more frequently when an intraoperative navigation system is applied [194]. Even if an improved orientation could theoretically reduce the complication rates, for which there are some indications in retrospective studies [195], no trial has to date been published that could convincingly confirm such an effect [196]. Because of the low complication rates of FESS, demonstrating differences in complication rates is rather difficult [196], requiring very large multicentre studies.

On the other hand, the application of navigation systems appears to reduce the mental stress on the surgeon [197], [198], thereby creating better preconditions to lower the complication rates. Reduced failure rates were found, for example, in a laparoscopic model where the surgeons experienced less mental distress [199].

##### 3.2.4.5 Postoperative treatment after FESS

Follow-up treatment after FESS is an important part of achieving therapeutic success. To date, a series of different procedures on postoperative follow-up have been suggested. In a meta-analysis, Rudmik et al. [200] evaluated seven different procedures of early postoperative care (up to 12 weeks postoperatively). Based on the results of this meta-analysis, rinsing with saline solution, suction of the paranasal sinuses, and topical steroids are recommended for early postoperative treatment.

In contrast to this procedure, postoperative antibiotic treatment and the application of systemic or non-standardized steroids, for example, in steroid-coated stents, are discussed as options by Rudmik et al., and may be indicated under certain circumstances [200]. However, routine application in the postoperative follow-up is not recommended.

The application of topical steroids after FESS was investigated in several prospective randomised trials. Fandino and co-workers summarised them in a meta-analysis in 2013 [201]. The evaluation of 11 RCTs with 945 patients revealed that the application of topical steroids in the first postoperative year reduced the subjective symptoms of the affected patients and that recurrence of polyps was significantly decreased. Consequently, the use of topical steroids after FESS was recommended.

In a Cochrane analysis from 2015, studies were analysed that applied steroid-releasing stents after FESS [202]. From an initial 159 studies, 21 were identified that were eligible for a meta-analysis. However, because of the strict inclusion and exclusion criteria of the Cochrane analysis, finally no study was included. Therefore, the authors concluded that currently no statement could be given regarding the efficacy of applying steroid-releasing stents and that in this context, high-quality randomised prospective trials are required.

Not only is the postoperative follow-up after FESS not standardised, the question as to whether nasal packing should be applied can still not be answered by high-quality studies. Even when nasal packing should be applied, it is unclear whether absorbable or non-absorbable materials should be used. In 2015, Wang et al. concluded from a current literature research and meta-analysis of five studies with 241 operated paranasal sinuses that there was some evidence of better tolerance of absorbable packings, but that overall several studies had contradictory results and thus no definite recommendation for a certain type of packing was given [203].

A prospective randomised trial conducted by Franklin and Wright came to the conclusion that patient satisfaction after the application of absorbable packings was clearly greater compared to non-resorbable packings that had to be removed [204], whereas the endoscopic results were similar. Kastl et al. demonstrated an advantage of absorbable packings in comparison with not applying any packings [205].

## 4 Summary and outlook

In this review, an up-to-date overview of the recent literature on the treatment of nasal obstruction and rhinosinusitis is presented. Even though the symptoms and the treatments are sometimes very similar, the recommendations based on the current literature are of various grades because the available studies have greatly varying evidence levels. In particular, the treatment of chronic nasal obstruction caused by deformities of the inner or outer nose is not based on good evidence, because the performance of surgical studies with high evidence levels has to cope with numerous challenges that currently can only be resolved theoretically. A consensus on internationally accepted standards for the definition of basic pathologies as well as appropriate outcome parameters is crucial for future trials.

## Abbreviations

- AERD: aspirin-exacerbated respiratory disease
- AIS: analgesics intolerance syndrome
- ARS: acute rhinosinusitis
- ASA: acetylsalicylic acid
- AWMF: Association of the Scientific Medical Societies in Germany (Arbeitsgemeinschaft der Wissenschaftlichen Medizinischen Fachgesellschaften e.V., AWMF)
- CRS: chronic rhinosinusitis
- EbM: evidence-based medicine
- EPOS: European position paper on rhinosinusitis and nasal polyps
- FESS: functional endoscopic sinus surgery
- GRADE: grading of recommendations assessment, development, and evaluation
- OCEBM: Oxford Centre for Evidence-Based Medicine
- RCT: randomised controlled trial

## Notes

### Acknowledgements 

I wish to give my very special thanks to Prof. M. Scheithauer for his valuable comments and for the figures on turbinate surgery. Furthermore, I thank Mrs. Vazquez and Mrs. Reith for their tremendous support regarding the literature research.

### Competing interests

The author declares that she has no competing interests.

## Figures and Tables

**Table 1:**
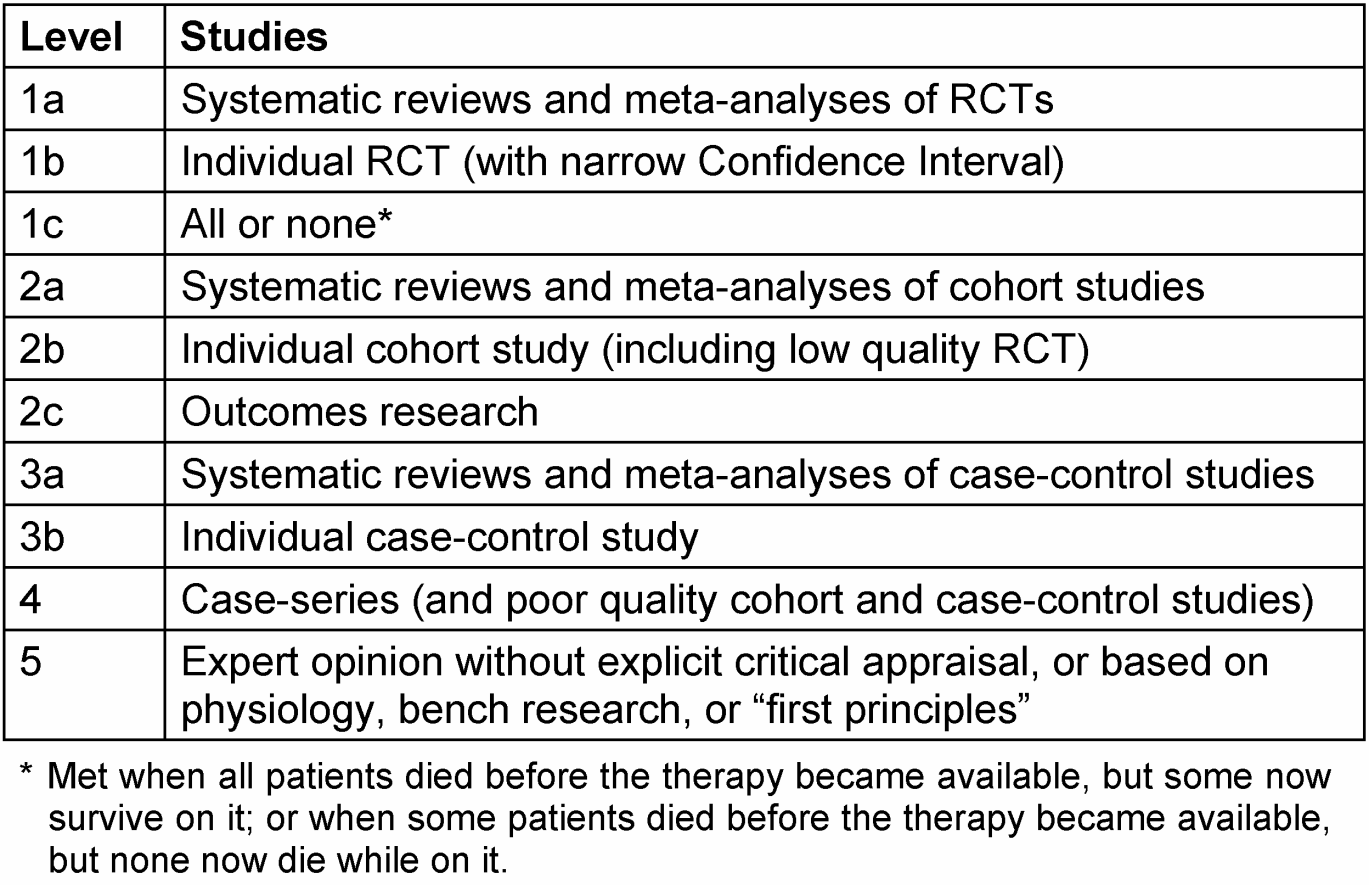
Levels of evidence according to OCEBM, updated in 2009 [205]

**Table 2:**
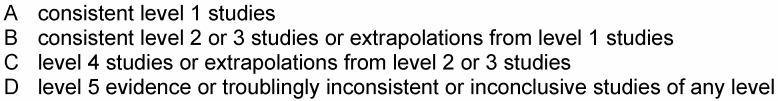
Grades of recommendation of the 2009 Oxford levels of evidence

**Table 3:**
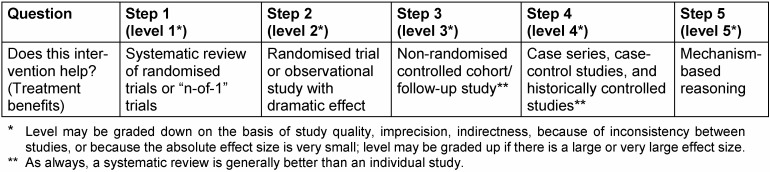
Levels of evidence according to OCEBM, the Oxford 2011 levels of evidence [206]

**Table 4:**
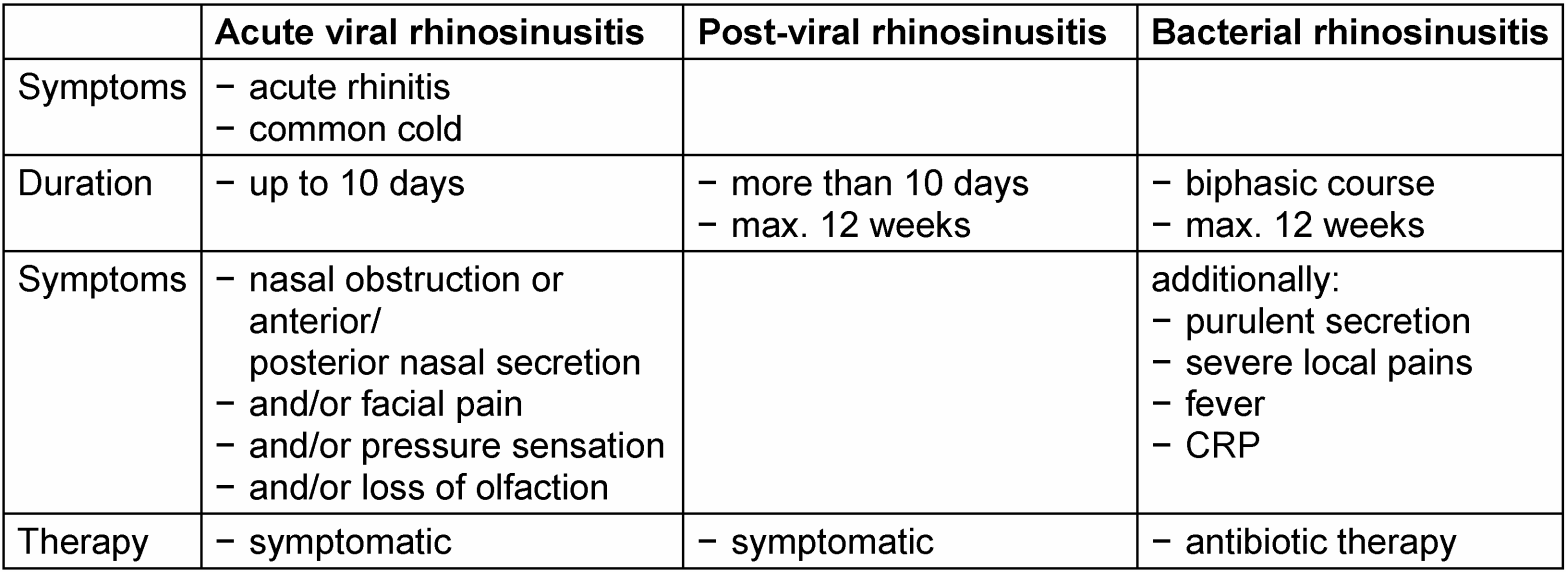
Types and characteristics of acute rhinosinusitis

**Table 5:**
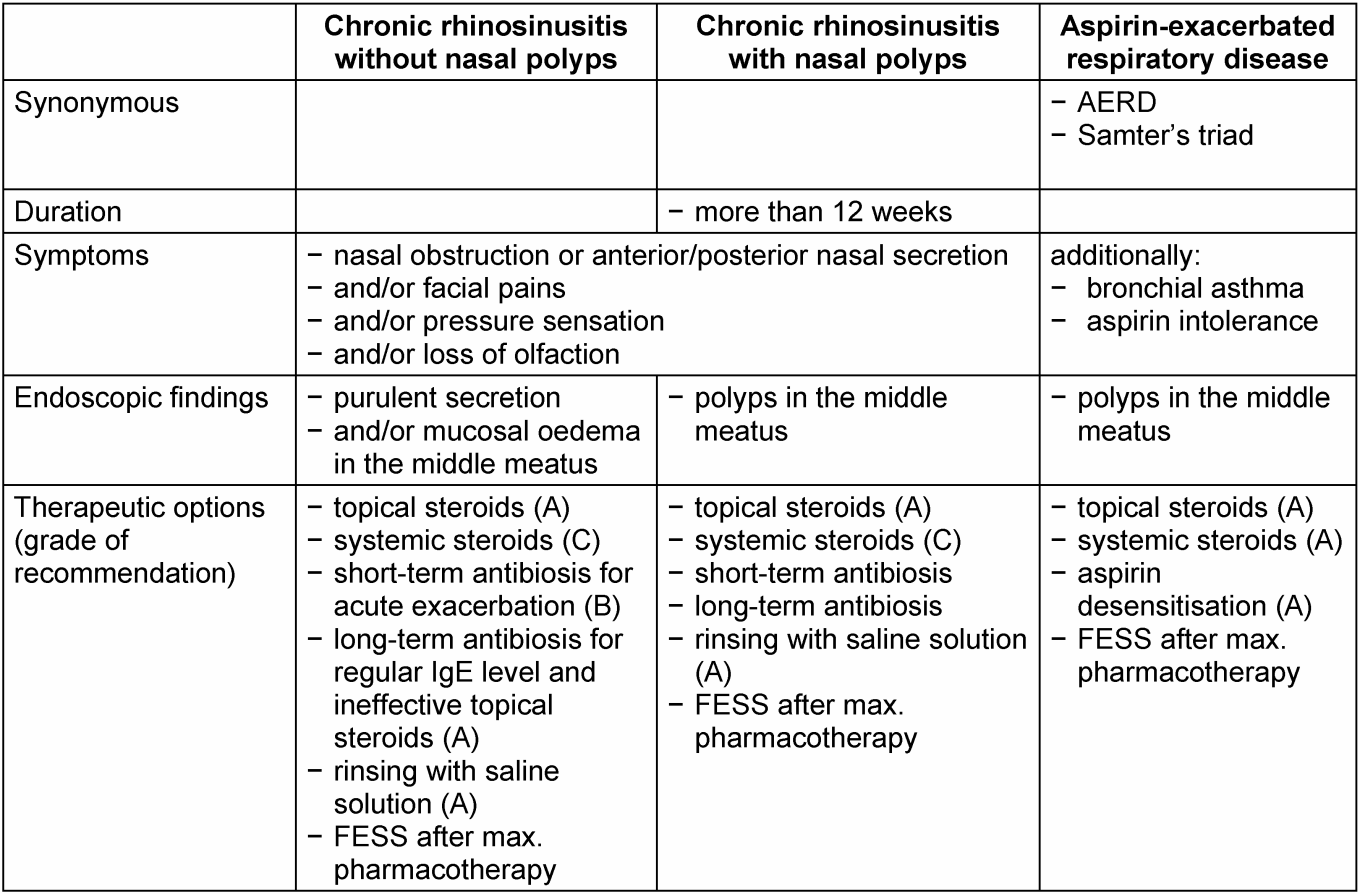
Types and characteristics of chronic rhinosinusitis
